# Case Report: CD19 CAR-T therapy induces durable remission in a pediatric patient with TP53-mutated, refractory Burkitt lymphoma: a 30-month follow-up

**DOI:** 10.3389/fonc.2026.1702660

**Published:** 2026-01-20

**Authors:** Yong Zhou, Xuerong Shen, Qinwei Chen, Yuping Guo, Dongyan Shen, Rui Su

**Affiliations:** 1 Department of Hematology, The First Affiliated Hospital of Xiamen University, School of Medicine, Xiamen University, Xiamen, China; 2 Department of Internal, Shanghang County Hospital, Longyan, China; 3 Department of Xiamen Cell Therapy Research Center, The First Affiliated Hospital of Xiamen University, School of Medicine, Xiamen University, Xiamen, China

**Keywords:** adolescent and young adult, Burkitt’s lymphoma, chimeric antigen receptor T-cell, refractory/relapsed, TP53 mutations

## Abstract

Burkitt lymphoma (BL) with TP53 mutations is characterized by strong chemoresistance and poor prognosis, posing significant challenges in clinical treatment. Chimeric antigen receptor T-cell (CAR-T) therapy has shown promise in refractory/relapsed (r/r) BL, but its efficacy in TP53-mutated cases remains to be further validated. This case report describes a 16-year-old male adolescent diagnosed with TP53-mutated BL, whose initial presentation with recurrent abdominal pain led to a misdiagnosis of high-grade B-cell lymphoma. The disease was refractory to two cycles of chemotherapy (DA-EPOCH-R and AZA + R-CDOP) and even progression after third-line R-CODOX-M therapy. Following reevaluation confirming BL, the patient received CD19-directed CAR-T cell therapy (Axicabtagene Ciloleucel infusion, Axi-cel) and achieved complete metabolic response (CMR). Baseline lactate dehydrogenase (LDH) was markedly elevated at 1,343 U/L. As of the latest follow-up in March 2025, the patient remains in remission at 30 months after CAR-T infusion with full functional recovery, including resumption of normal academic life. This case, among the youngest reported uses of commercial Axi-cel for BL, highlights the diagnostic complexity in adolescent lymphoma and demonstrates that CD19 CAR-T therapy can overcome TP53-associated chemoresistance in adolescent BL. It also suggests that integrating molecular profiling and immunotherapy may provide new strategies for managing high-risk, treatment-refractory cases in the adolescent and young adult population.

## Introduction

Burkitt lymphoma (BL) is a highly aggressive B-cell non-Hodgkin lymphoma (NHL), typically manifesting in both adults and children. It is characterized by extra-nodal involvement, rapid tumor progression, and high mortality rates, driven primarily by hyperproliferation resulting from MYC oncogene translocation (1). While accounting for 30%-50% of pediatric non-Hodgkin lymphomas, BL in the adolescent and young adult (AYA) population (15–39 years) often exhibits more aggressive features than in younger children, particularly when associated with TP53 mutations (2). TP53, located on chromosome 17p, encodes the p53 tumor-suppressor protein, playing a fundamental role in DNA repair, cell cycle arrest, apoptosis, senescence, and autophagy (3). In BL, TP53 aberrations, including missense and nonsense mutations, are associated with complex karyotypic instability, impaired cell cycle regulation, and rapid disease progression. Patients harboring TP53 mutations demonstrate remarkable resistance to standard immunochemotherapy regimens (e.g., DA-EPOCH-R and CODOX-M/IVAC), reflecting a dichotomous prognostic landscape. Those cases with wild-type TP53 exhibit outcomes that vary with mutation burden, whereas poor prognosis of TP53-mutated cases is notable regardless of coexisting molecular alterations (4). This dichotomy is particularly distinct in AYA patients, who carry higher TP53 mutation frequencies (35%-45%) versus pediatric BL (15%-20%) (5).

Thus, the AYA population frequently falls into a treatment gap between pediatric and adult protocols, with limited data to guide therapy for the high-risk cases. This report describes a case of TP53-mutated Burkitt lymphoma (BL) in an adolescent, who experienced multiple lines of treatment failure and ultimately achieved sustained remission through CAR-T cell therapy. Accordingly, diagnostic and therapeutic approaches for this high-risk population were explored, and the clinical utility of CAR-T therapy was assessed. This case report highlights a potential approach in managing such cases, showing that CAR-T cell therapy can serve as an effective treatment option for achieving sustained remission in this challenging patient group.

## Case presentation

**Figure 1:**
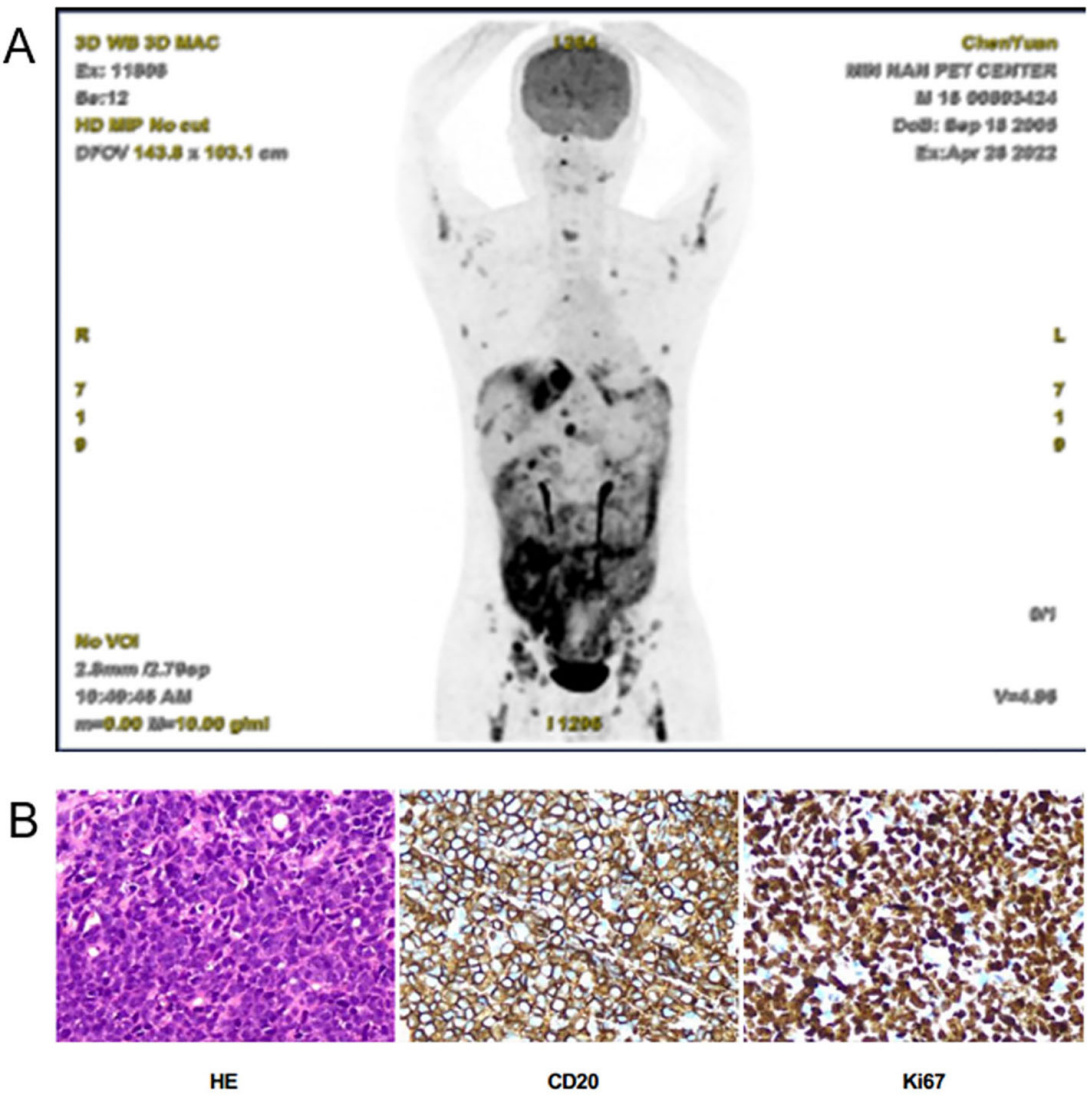
Preliminary diagnostic findings upon admission. **(A)** PET-CT displays hypermetabolic lesions, involving mediastinal lymph nodes, peritoneal soft tissue masses, and multiple osseous lesions with splenomegaly but no central nervous system involvement, indicating lymphoma. **(B)** Peritoneal biopsy confirmed high-grade B-cell lymphoma.

A 16-year-old previously healthy boy was admitted on April 24, 2022, with a 2-week history of recurrent epigastric pain (unresponsive to Spasmomen and omeprazole) and acute postprandial vomiting, fever (38.9 °C), chills, and dry mouth 2 h prior to admission. Initial laboratory tests showed markedly elevated lactate dehydrogenase (1,343 U/L); other blood tests showed normal results. Contrast-enhanced CT revealed bilateral pulmonary infiltrates, minimal pleural effusions, diffuse ascites, and suspected cholecystitis. Abdominal ultrasound identified a 7.8×2.4 cm enteric fistula. PET-CT demonstrated hypermetabolic lesions in mediastinal lymph nodes (SUVmax 6.6), peritoneal soft tissue masses (SUVmax 7.3), and multiple osseous lesions (SUVmax 7.7) with splenomegaly (no CNS involvement) (**Figure 1A**). Ascitic fluid analysis detected 9.2% CD19^+^ abnormal B cells (loss of surface light chain). Peritoneal biopsy confirmed high-grade B-cell lymphoma, NOS (HGBL, NOS) with immunophenotype CD20^+^/CD19^+^/CD10^+^/BCL6^+^, and Ki-67 >90%. Flow cytometry showed a CD5^−^/CD10^+^ large B-cell phenotype. The initial MYC FISH results were negative, but molecular testing identified a TP53 mutation (no other recurrent BL-associated mutations such as ID3, TCF3, or CCND3 were detected). Bone marrow examination revealed 10.5% abnormal lymphocytes and 1.1% CD5^−^/CD10^+^/CD19^+^/CD20^+^ monoclonal B-cell infiltration (**Figure 1B**). The final diagnosis was stage IVB HGBL, NOS (aaIPI score 3) with intra-abdominal infection, and periappendiceal abscess.

**Figure 2:**
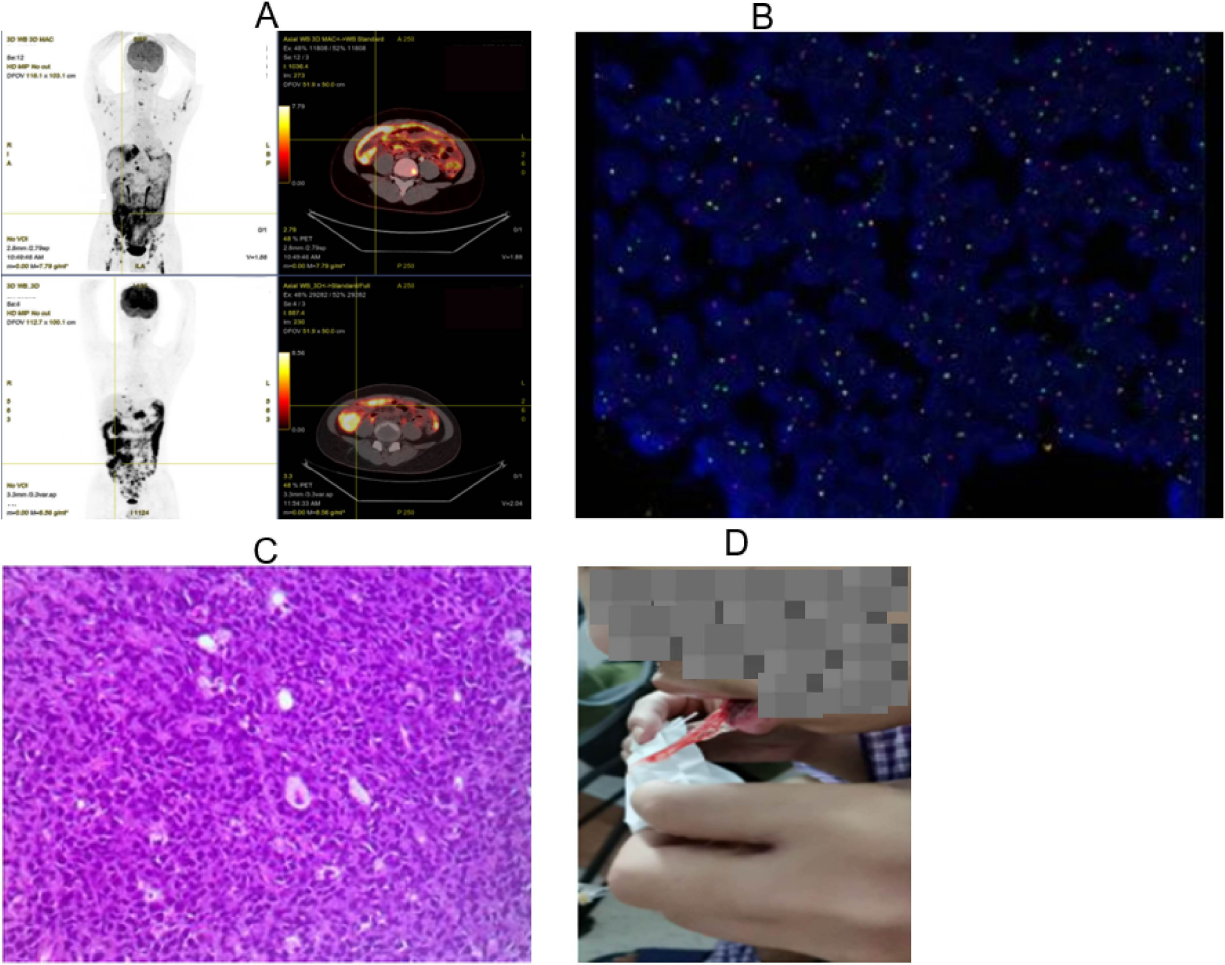
Imaging and pathological evidence of disease progression following conventional chemotherapy. **(A)** Posttreatment PET-CT displays disease progression, with worsening peritoneal involvement despite resolution of previously hypermetabolic osseous lesions and partial regression of mediastinal lymphadenopathy. **(B)** Posttreatment FISH demonstrates MYC rearrangement (41.5% positivity). **(C)** Histopathological examination of the repeat peritoneal biopsy confirmed Burkitt lymphoma (TP53-mutated, Stage IVB, high-risk). **(D)** The patient experienced significant oral mucosal hemorrhage after the definitive diagnosis of Burkitt lymphoma and subsequent two cycles of chemotherapy.

Per NCCN guidelines (6), the patient received CTX+DXM for tumor debulking, followed by dose-reduced DA-EPOCH-R 3 days later due to periappendiceal abscess. On May 28, 2022, second-line AZA+R-CDOP was initiated for persistent abscess and TP53 mutation. Post-2-cycle evaluation showed progressive disease (PD) on PET-CT: Peritoneal SUVmax increased from 7.3 to 13.5, with new abdominopelvic lymphadenopathy and worsening ascites (osseous lesions resolved, mediastinal lymphadenopathy partially regressed) (**Figure 2A**). CNS involvement was excluded.

For disease progression, repeat peritoneal biopsy was performed. While awaiting results, CAR-T preparation (lymphocyte collection on July 2) was initiated per ZUMA-12 criteria for refractory disease. On July 10, definitive diagnosis of TP53-mutated Burkitt lymphoma (BL, stage IVB, high-risk) was confirmed with MYC rearrangement (41.5% by FISH) (**Figure 2B**), immunophenotype CD20^+^/CD19^+^/CD5^−^/CD10^+^/BCL6^+^/BCL2^−^, MYC expression (80%), p53 expression (90%), and Ki-67 >90% (persistent intra-abdominal infection/abscess) (**Figure 2C**).

The diagnosis of BL was supported by findings from the repeat peritoneal biopsy, which demonstrated a CD20^+^/CD19^+^/CD5^−^/CD10^+^/BCL6^+^/BCL2^−^ immunophenotype, MYC protein expression in approximately 80% of tumor cells, p53 overexpression in 90%, and a very high proliferation index (Ki-67 >90%). Cytogenetic analysis confirmed MYC rearrangement by FISH in 41.5% of nuclei. These features, in conjunction with clinical and imaging characteristics, led to the reclassification from high-grade B-cell lymphoma, NOS to TP53-mutated BL.

Third-line R-CODOX-M caused grade 2 hepatotoxicity, grade 4 myelosuppression, and oral mucositis. Fourth-cycle R-HyperCVAD-A on August 21, 2022, led to profound myelosuppression, refractory thrombocytopenia, and mucosal hemorrhage (**Figure 2D**), with subsequent confirmation of PD. The multidisciplinary team deemed the disease chemotherapy-refractory and proceeded with CAR-T therapy. The protocol was approved by the Ethics Committee of The First Affiliated Hospital of Xiamen University (No.: 2022-004).

**Figure 3:**
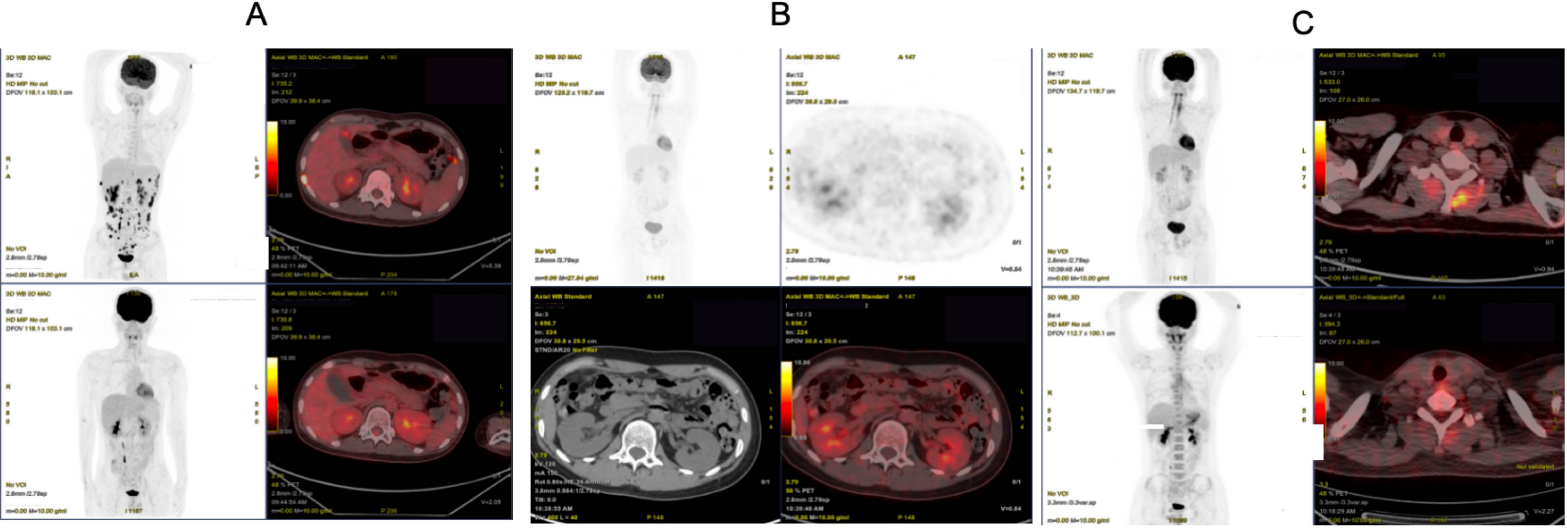
Efficacy evaluation of CAR-T cell therapy. **(A)** PET/CT performed 1 month after Car-T cell infusion demonstrated a complete metabolic response. **(B)** PET/CT performed 3 months after CAR-T cell infusion indicated a complete metabolic response. **(C)** PET/CT performed 8 months after CAR-T cell infusion revealed a complete metabolic remission.

**Figure 4:**
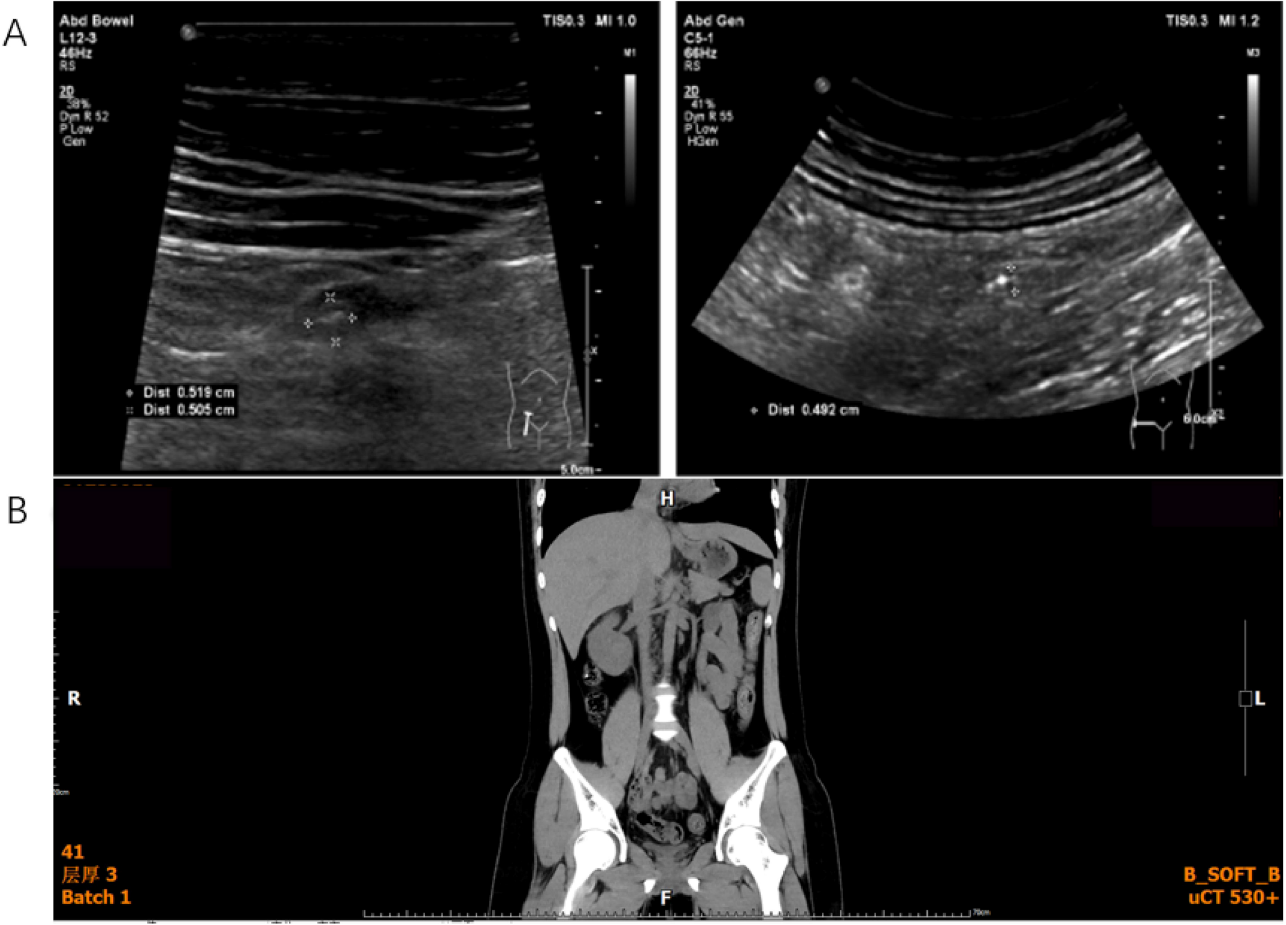
Ultrasonography **(A)** and CT **(B)** of the abdomen showed no signs of recurrence.

Pre-CAR-T assessment showed a palpable right abdominal mass, normal organ function, and no contraindications. Lymphodepletion with FC regimen was administered on September 16, followed by Axicabtagene Ciloleucel injection (Axi-cel) infusion on September 20 (total dose: 2.0 × 10^6^ CAR^+^ T cells/kg) (7). Adverse events included grade 1 CRS (fever, day 3), localized CRS (abdominal distension, day 8), and grade 4 ICANS (trismus, upward gaze, seizures, day 11) (8), all managed successfully. Response assessment showed CMR on 1-month PET-CT (October 20) (**Figure 3A**). PD-1 inhibitor maintenance (200 mg q4w) with IVIG was initiated on November 23. Follow-up PET-CT showed Deauville score 2–3 (3 months, December 20) (**Figure 3B**) and sustained CMR (9 months, May 2023) (**Figure 3C**). As of March 2025, ultrasonography and CT showed no signs of recurrence with full functional recovery (**Figure 4**), including resumption of academic life.

## Discussion

This case report presents a compelling example of how CAR-T therapy can overcome intrinsic chemoresistance in a high-risk adolescent with TP53-mutated BL, achieving a durable CMR lasting over 30 months. As one of the youngest reported patients to receive commercial Axi-cel for BL and the first to demonstrate such prolonged remission in the context of TP53 mutation, this case highlights the unique value of well-documented, real-world clinical observations in advancing therapeutic strategies for rare and refractory malignancies.

The clinical trajectory of this patient underscores several critical challenges in the management of AYA lymphoma. First, the initial misdiagnosis as high-grade B-cell lymphoma, NOS, due to negative MYC FISH and absence of classic morphological review, illustrates the diagnostic complexity in this age group, where overlapping features between BL and other aggressive B-cell lymphomas may lead to therapeutic delays. Second, the presence of a TP53 mutation—a well-established marker of poor prognosis in BL—was associated with rapid progression despite multiple intensive chemotherapy regimens, including DA-EPOCH-R, AZA + R-CDOP, and R-CODOX-M/R-HyperCVAD (9). This profound chemoresistance aligns with growing evidence that TP53 aberrations confer resistance to DNA-damaging agents, rendering conventional cytotoxic therapies ineffective and underscoring the urgent need for alternative, mechanism-driven approaches.

The successful outcome following CD19-directed CAR-T cell therapy (Axi-cel) in this case is particularly significant. Emerging evidence suggests that CD19-guided CAR-T cell therapy induces remission of refractory or recurrent BL, including in adult and pediatric cases (10), supporting the decision to adopt this treatment approach. Unlike chemotherapy, which relies on intact p53-mediated apoptosis, CAR-T cells exert their cytotoxic effects through granzyme/perforin and death receptor pathways, independent of TP53 status (11). This p53-independent mechanism likely enabled the eradication of TP53-mutated tumor cells that had proven refractory to all prior therapies. Moreover, emerging translational evidence suggests that sustained CAR-T cell persistence and resistance to exhaustion are critical determinants of durable remission, as demonstrated by recent mechanistic studies showing that modulation of intrinsic inhibitory pathways can enhance cytotoxicity, proliferative capacity, and long-term *in vivo* persistence of engineered T cells (12, 13). Notably, despite the development of grade 4 immune effector cell-associated neurotoxicity syndrome (ICANS), the toxicities were manageable with prompt intervention, supporting the feasibility of CAR-T therapy even in complex clinical settings with active infection and organ compromise.

Additionally, the sustained remission and full functional recovery—including return to academic life—highlight the potential for meaningful quality-of-life improvement with cellular therapy, a dimension often underreported in large registries. The decision to administer the PD-1 inhibitor as maintenance therapy was based on institutional experience and emerging preclinical data, suggesting that immune checkpoint inhibition may mitigate T-cell exhaustion and enhance persistence of CAR-T cells in the tumor microenvironment. This approach was not protocol-driven but rather an off-label, empiric strategy used in high-risk lymphomas with concerns about early relapse or suboptimal CAR-T expansion. While no direct clinical trials support PD-1 blockade as post-CAR-T maintenance in Burkitt lymphoma, the safety profile of sintilimab has been established in other B-cell malignancies, including a phase 1/2 trial (NCT03769181) (14), which demonstrated manageable toxicity without new safety signals at this dose. Notably, the patient tolerated the regimen well, with no immune-related adverse events observed during or after treatment. The contribution of PD-1 inhibition to durable remission remains speculative and warrants further investigation in prospective studies.

This case reinforces the role of CAR-T cell therapy as a potentially curative salvage option for AYA patients with TP53-mutated, chemotherapy-refractory BL. It demonstrates the power of individualized, biomarker-guided immunotherapy in overcoming high-risk genetic features and fills a critical evidence gap for a patient population often falling between pediatric and adult treatment paradigms. As cellular therapies become more accessible, such meticulously documented cases will continue to inform clinical decision-making, guide trial design, and ultimately expand the therapeutic horizon for rare and aggressive cancers.

## Data Availability

The original contributions presented in the study are included in the article/supplementary material. Further inquiries can be directed to the corresponding authors.
